# L-asparaginase is a PAR2 N-terminal protease that unmasks the PAR2 tethered ligand

**DOI:** 10.1038/s41420-025-02467-z

**Published:** 2025-04-08

**Authors:** Jung Kwon Lee, Karl Riabowol, Xidi Wang, Ki-Young Lee

**Affiliations:** 1 Arnie Charbonneau Cancer and Alberta Children’s Hospital Research Institutes, Department of Biochemistry and Molecular Biology, University of Calgary, Calgary, AB Canada; 2 Arnie Charbonneau Cancer and Alberta Children’s Hospital Research Institutes, Department of Cell Biology & Anatomy, University of Calgary, Calgary, AB Canada; 3 Arnie Charbonneau Cancer and Alberta Children’s Hospital Research Institutes, Department of Oncology, University of Calgary, Calgary, AB Canada; 4 Central Laboratory of the Medical Research Center, The First Affiliated Hospital of Ningbo University, Ningbo, PR China

**Keywords:** Acute lymphocytic leukaemia, Acute lymphocytic leukaemia

## Abstract

L-asparaginase is an indispensable chemotherapeutic drug for patients with acute lymphoblastic leukemia (aLL), a life-threatening lymphoid neoplasm and the prime cause of cancer death among children. Previously, we reported that L-asparaginase kills aLL cells via an excessive rise in [Ca^2+^]_i_ due to IP3R-mediated ER Ca^2+^ release followed by stimulation of the intrinsic apoptotic pathway (Blood, 133, 2222-2232). We also demonstrated that L-asparaginase triggers ER Ca^2+^ release by targeting the G-protein-coupled receptor (GPCR), protease-activated receptor 2 (PAR2) (Cell Death & Discovery, 10:366). However, how L-asparaginase stimulates PAR2 remains unknown. Here, we show that elastase, which can disarm trypsin-mediated PAR2 activation by cleaving a S_67_-V_68_ residue downstream of the tethered ligand (TL) and removing it from PAR2, abrogates L-asparaginase-induced ER Ca^2+^ release, indicating that L-asparaginase targets the TL-containing PAR2 N-terminal extracellular domain to induce ER Ca^2+^ release. Inactive forms (T_111_V/K_184_T or D_112_T/K_184_T) of L-asparaginase do not induce ER Ca^2+^ release in μ-opioid receptor 1 (µ-OR1)-knockdown aLL cells, suggesting that L-asparaginase action on PAR2 requires its enzymatic activity. Time-lapse confocal microscopy of cells expressing mRFP-hPAR2-eYFP and nanoluciferase (Nluc) reporter release assays of cells expressing Nluc-hPAR2-eYFP showed that L-asparaginase cleaves PAR2 at the N-terminal extracellular I_26_-G_71_ domain. Cleavage assay of a PAR2 N-terminal peptide by L-asparaginase and subsequent LC-MS/MS analysis show that L-asparaginase is a PAR2 protease that cleaves N_30_-R_31_ and R_31_-S_32_ residues, unmasking the PAR2 TL. Thus, our findings reveal for the first time the molecular mechanism through which L-asparaginase activates PAR2, leading to perturbation of intracellular Ca^2+^ homeostasis and aLL cell apoptosis.

## Introduction

Acute lymphoblastic leukemia (aLL) is a dreadful cancer in the bone marrow and blood characterized by the expansion of immature B- (in ∼85% of cases) and T- (in ∼15% of cases) lymphocytes [1]. It is also the most widespread pediatric cancer and the leading cause of cancer death among children [2]. L-asparaginase is an enzyme chemotherapeutic drug that has been used for the treatment of aLL since the late 1960s [3–5].

L-asparaginase kills aLL cells by activating the intrinsic apoptotic pathway [6]. We have shown this by demonstrating that L-asparaginase-induced IP3R-mediated ER Ca^2+^ release triggers perturbation of intracellular Ca^2+^ homeostasis and subsequently upregulation of the Ca^2+^-mediated calpain-1-Bid-caspase-3/12 apoptotic pathway [6]. Ca^2+^ released from the ER is transferred into mitochondria, elevating mitochondrial Ca^2+^ and reactive oxygen species (ROS) levels [7] that cause mitochondrial permeability transition pore (mPTP) formation leading to a rise in [Ca^2+^]_cyt_ and aLL cell apoptosis [7]. Indeed, inhibition of ER-mitochondria Ca^2+^ transfer by Ruthenium red [RuR; a mitochondrial calcium uniporter (MCU) inhibitor], mitochondrial ROS production by Mito-Tempo, and/or mPTP formation by cyclosporine A (CsA) blocks L-asparaginase-induced apoptosis [7]. Recently, we also showed that L-asparaginase targets two different G-protein-coupled receptors (GPCRs), μ-opioid receptor 1 (µ-OR1; also known as OPRM1 or MOR1) [8] and protease-activated receptor 2 (PAR2) [9], and induces IP3R-mediated ER Ca^2+^ release in aLL cells [6] via µ-OR1-G_αi_-AC-↓[cAMP]_i_-↓PKA-↓pSer1105-PLCβ3 and PAR2-G_αq_-PLCβ3 pathways [9].

PAR2 consisting of three domains: i.e., an extracellular N-terminal domain, a transmembrane domain with seven pseudo-parallel helices connected by three extracellular loops (ECL 1, 2 & 3) and three intracellular loops, and an intracellular C-terminal domain [10] is activated by the serine protease, trypsin, and other proteolytic enzymes [11]. PAR2 cleavage by trypsin exposes the N-terminal S_37_LIGKV_42_ (aka tethered ligand: TL) that folds back and self-activates PAR2 by binding to conserved regions of ECL2 [11]. PAR2 activation induces IP3R-mediated ER Ca^2+^ release and is involved in regulating diverse cellular processes [12]. However, how L-asparaginase activates PAR2, which also induces IP3R-mediated ER Ca^2+^ release, remains undefined.

L-asparaginase is well known for its amidohydrolase (EC 3.5.1.1) activity that utilizes H_2_O and acts on the α-carbon side chain amide bond of L-asparagine, resulting in the deamidation of L-asparagine and formation of L-aspartate [13]. However, L-asparaginase also has β-aspartyl peptidase activity [13–18]. Under physiological conditions, asparaginyl peptide can be deaminated to form a cyclic imide intermediate that when hydrolyzed, forms a β-aspartyl peptide. Thus, L-asparaginase has the ability to cleave the asparaginyl peptide by targeting the β-linked aspartic acid residue and creating a new N-terminal end of a polypeptide, resulting in formation of two shorter peptides. Interestingly, the PAR2 extracellular N-terminal domain (I_26_-G_71_; M_1_-T_25_ is a signal peptide) harbors an asparagine (N_30_) which is located just upstream of the TL, indicating that it is an asparaginyl peptide that can form a β-aspartyl peptide. Thus, it is possible that L-asparaginase may target the N_30_ residue, exposing the TL (S_37_LIGKV_42_) which then can self-activate PAR2 via ECL2 binding. However, this awaits further investigation.

In this study, we utilize single-cell Ca^2+^ imaging, small molecular agonists and inhibitors, knockdown strategies, time-lapse confocal microscopy of cells expressing mRFP-hPAR2-eYFP and Nluc reporter release assays of cells expressing Nluc-hPAR2-eYFP, and the synthetic PAR2 N-terminal peptide cleavage by L-asparaginase and subsequent LC-MS/MS analysis to elucidate the molecular mechanism by which L-asparaginase stimulates PAR2. We show here that L-asparaginase cleaves two distinct sites, N_30_-R_31_ and R_31_-S_32_, that could expose the PAR2 TL, eliciting IP3R-mediated ER Ca^2+^ release in aLL cells, with this perturbation leading to aLL cell apoptosis.

## Results

### L-asparaginase, which induces IP3-mediated ER Ca^2+^ release [6, 7, 19], targets the TL-containing PAR2 N-terminal extracellular domain

**Fig. 1:**
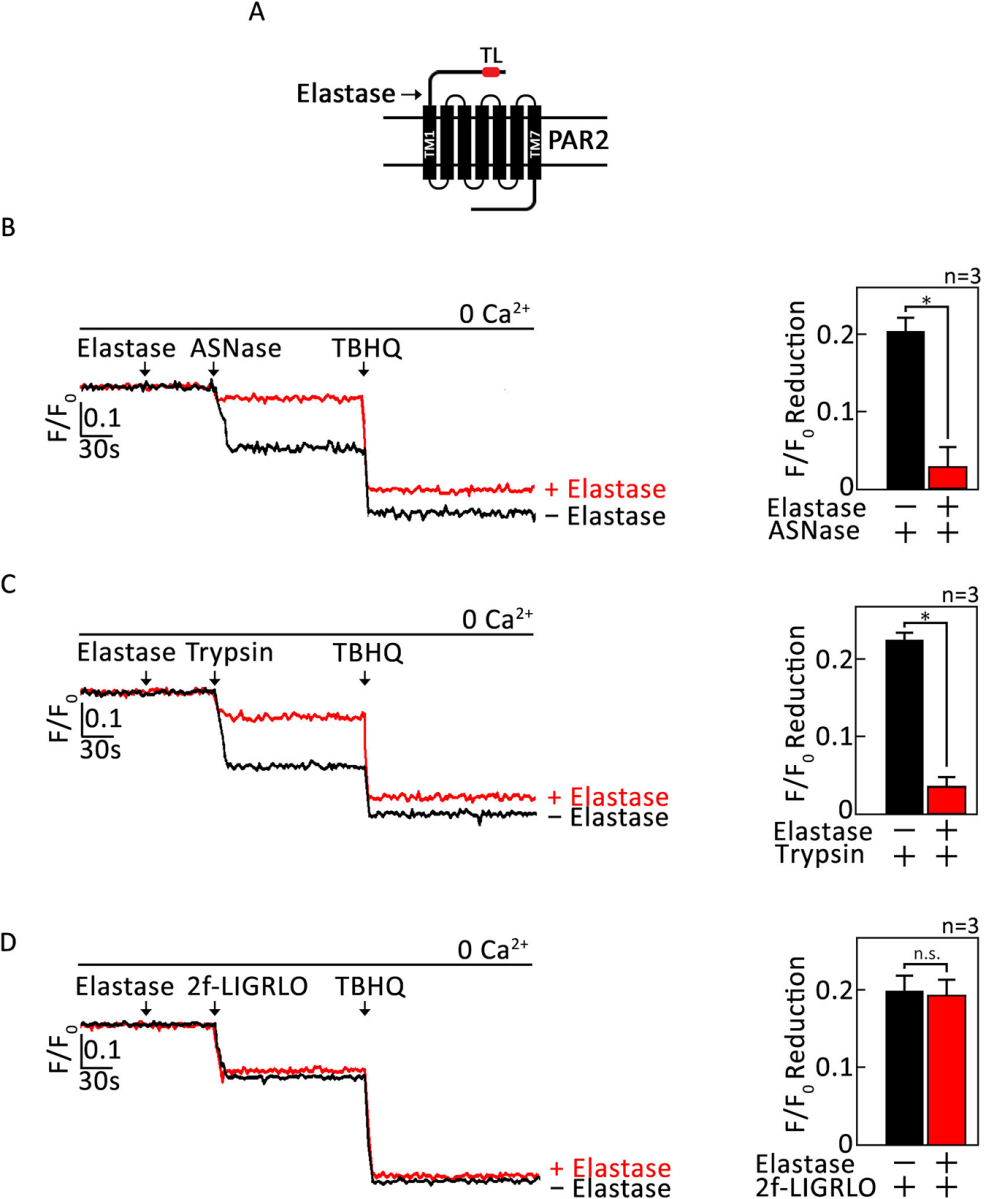
Pretreatment with elastase abrogates L-asparaginase-induced ER Ca^2+^ release. **A** Schematic diagram of cell membrane PAR2. N-terminal extracellular domain (I_26_-G_71_) [32], seven transmembrane domains (black bars 1–7), three extracellular loops, three intracellular loops and an intracellular C-terminal domain were shown. The arrow is directed at the elastase cleavage site, S_67_-V_68_, downstream of the tethered ligand (TL; the red-colored box in the N-terminal extracellular domain). **B**–**D** SEM cells loaded with Mag Fluo-4 AM were subjected to Ca^2+^ tracing by single-cell Ca^2+^ imaging. After obtaining stable baseline ER Ca^2+^ levels for 1 min, cells were pretreated (+; or not pretreated, −) with elastase for 1 min were exposed to L-asparaginase (ASNase; **B**), trypsin (**C**) or a PAR2 agonist 2-furoyl-LIGRLO-NH_2_ (2f-LIGRLO [21]; **D**) for 2 min to analyze ER Ca^2+^ release. The left panel shows the average Ca^2+^ tracing measured every two seconds in 20 individual cells after ASNase (**B**), trypsin (**C**) or 2f-LIGRLO (**D**) treatment. Treatment with TBHQ, an ER Ca^2+^ pump inhibitor, caused further ER Ca^2+^ release, indicating that these cells were viable during analysis. Data are from one of three independent experiments (*n* = 3) showing similar results. The charts on the right show the difference in ER Ca^2+^ release following treatment with ASNase (**B**), trypsin (**C**) or 2f-LIGRLO (**D**) pretreated (or not pretreated) with elastase. *F*/*F*_0_ value of 1 min after ASNase (**B**), trypsin (**C**) or 2f-LIGRLO (**D**) was used to determine *F*/*F*_0_ reduction. Values are means ± SEM of the three independent experiments *p < 0.05. N.S. not significant.

**Fig. 2:**
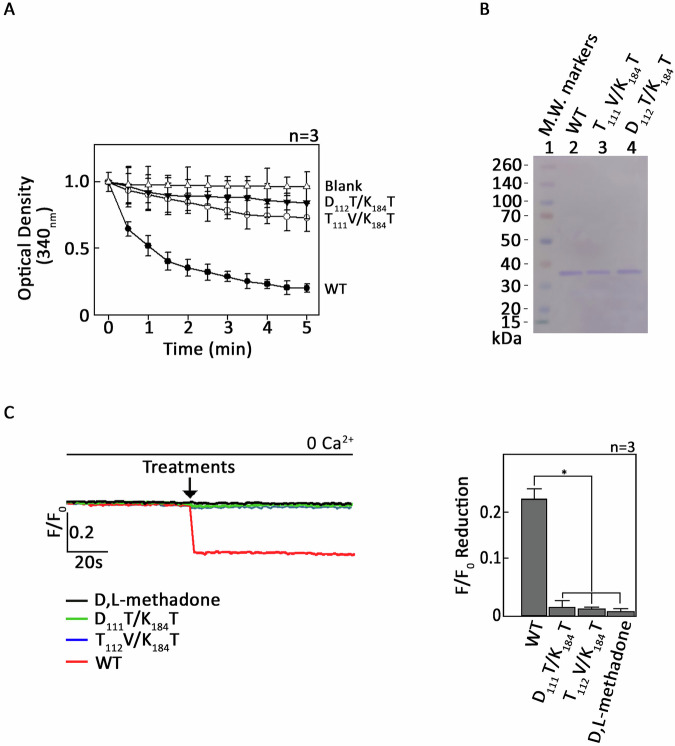
L-asparaginase stimulation of PAR2 requires its enzymatic activity. **A** L-asparaginase with T_111_V/K_184_T or D_112_T/K_184_T substitution displays minimal enzymatic activity. **B** wt (2 µg), T_111_V/K_184_T (1.5 µg) and D_112_T/K_184_T (2 µg) forms of L-asparaginase, purified as described in Materials and Methods, were resolved by 12.5% SDS-PAGE and stained with Coomassie brilliant blue R250. **C** µ-OR1-knockdown SEM cells loaded with Mag Fluo-4 AM were subjected to Ca^2+^ tracing by single-cell Ca^2+^ imaging. After obtaining stable baseline ER Ca^2+^ levels for 1 min, cells were treated with wt-, T_111_V/K_184_T- or D_112_T/K_184_T- form of L-asparaginase (0.5 µM) and analyzed for ER Ca^2+^ release. Data represent the average Ca^2+^ tracing per sec from 10 individual cells before and after treatment and are from one of three independent experiments (*n* = 3) showing similar results. The chart on the right shows the difference in *F*/*F*_0_ reduction, which corresponds to ER Ca^2+^ release following treatment. *F*/*F*_0_ values of 1 min wt-, T_111_V/K_184_T- or D_112_T/K_184_T- form of L-asparaginase was used to determine *F*/*F*_0_ reduction. Values are means ± SEM of the three independent experiments. *p < 0.05.

Elastase can disarm trypsin-mediated PAR2 activation by cleaving a S_67_-V_68_ residue downstream of the TL and removing it from PAR2 [20] (Fig. 1A). Thus, we tested whether L-asparaginase targets the TL-containing PAR2 N-terminal extracellular domain to elicit ER Ca^2+^ release. To do so, SEM aLL cells loaded with Mag Fluo-4-AM then pretreated (or not pretreated) with elastase were assessed for ER Ca^2+^ release following L-asparaginase treatment. As shown in Fig. 1B, elastase inhibited L-asparaginase-induced ER Ca^2+^ release, similar to its effect on trypsin (positive control; Fig. 1C) while having no effect on cells treated with the synthetic PAR2 agonist peptide, 2-furoyl-LIGRLO-NH_2_ (2f-LIGRLO) [21], which activates PAR2 without proteolytic TL exposure (negative control; Fig. 1D). This indicates that PAR2 activation by L-asparaginase likely occurs through TL exposure upon PAR2 N-terminal cleavage. Our data also suggest that L-asparaginase action requires its enzymatic activity. To test the latter possibility, catalytically inactive forms of L-asparaginase that carry T_111_V/K_184_T or D_112_T/K_184_T substitution, respectively [22–24] (Fig. 2A), were purified (Fig. 2B) and assessed for their inability to induce ER Ca^2+^ release in aLL cells lacking µ-OR1 [9]. The use of µ-OR1-knockdown SEM aLL cells is to detect only PAR2-induced IP3R-mediated ER Ca^2+^ release upon L-asparaginase treatment. As shown in Fig. 2C, both the T_111_V/K_184_T and D_112_T/K_184_T forms of L-asparaginase, that have negligible L-asparaginase activity, did not induce ER Ca^2+^ release, implying that L-asparaginase requires its enzymatic activity to stimulate PAR2-mediated ER Ca^2+^ release. Cells treated with wt L-asparaginase and D,L-methadone, a synthetic opioid that stimulates µ-OR1, serve as controls for L-asparaginase-induced PAR2-mediated ER Ca^2+^ release and lack of L-asparaginase-induced µ-OR1-mediated ER Ca^2+^ release, respectively.

### L-asparaginase cleaves the PAR2 N-terminal extracellular region

**Fig. 3:**
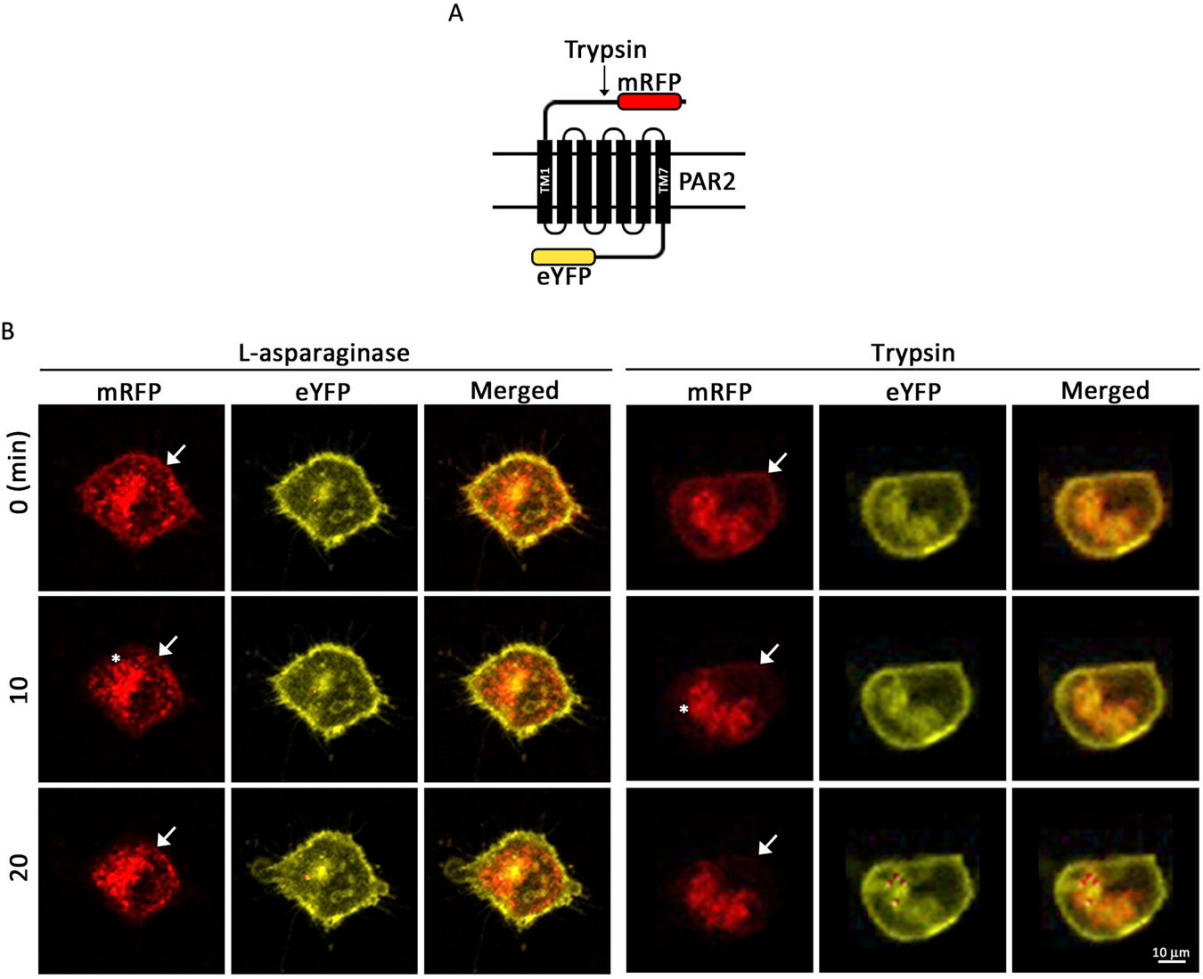
L-asparaginase-mediated cleavage of the PAR2 N-terminal extracellular domain releases mRFP from mRFP-PAR2-eYFP. **A** Schematic diagram of cell membrane mRFP-hPAR2-eYFP. mRFP is inserted into right after the PAR2 signal peptide (MRSPSAAWLLGAAILLAASLSCSGT) [25]. N-terminal extracellular domain (I_26_-G_71_) [32], seven transmembrane domains (black bars 1–7), three extracellular loops, three intracellular loops and an intracellular C-terminal domain were shown. eYFP is inserted into right before the PAR2 stop codon [25]. The arrow is directed at the trypsin cleavage site (R_36_-S_37_) in PAR2. **B** HEK293T cells expressing mRFP-PAR2-eYFP treated with L-asparaginase (left three panels) or trypsin (right three panels) were monitored by time-lapse confocal microscopy. Arrows and asterisks are directed at loss of mRFP signal at the cell periphery over time and PAR2 internalization to endosome-like structures observed after L-asparaginase and trypsin treatment, respectively.

To visualize L-asparaginase cleavage of PAR2 at the N-terminal extracellular domain, HEK293T cells stably transfected with pcDNA3.1(+) construct carrying monomeric red fluorescent protein (mRFP)-PAR2-enhanced yellow fluorescent protein (eYFP) [25] were subjected to N-terminal mRFP-release assay by time-lapse confocal microscopy (Fig. 3A). The dual tagging allowed us to detect PAR2 N-terminal cleavage; i.e., loss of mRFP signal while retaining eYFP signal. As shown in Fig. 3B, L-asparaginase reduced mRFP signal at the cell periphery over time, similar to cells treated with trypsin (positive control) while having no change in C-terminal reporter, eYFP signal.

**Fig. 4:**
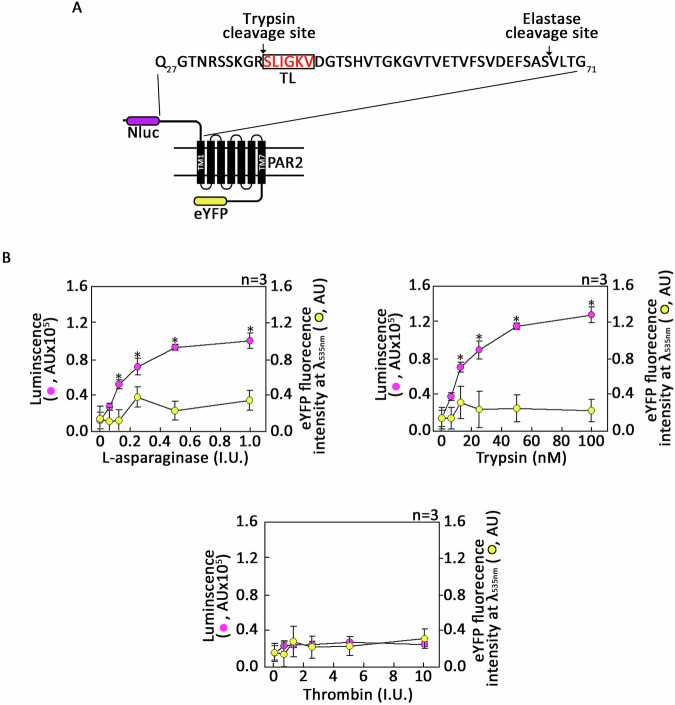
L-asparaginase-mediated cleavage of the PAR2 N-terminal extracellular domain releases Nanoluciferase (Nluc) from Nluc-hPAR2-eYFP. **A** Schematic diagram of cell membrane Nluc-hPAR2-eYFP [25]. Nluc is inserted into right after the PAR2 signal peptide (MRSPSAAWLLGAAILLAASLSCSGT) [25]. N-terminal extracellular domain (I_26_-G_71_) [32], seven transmembrane domains (black bars 1–7), three extracellular loops, three intracellular loops and an intracellular C-terminal domain are shown. eYFP is inserted into right before the PAR2 stop codon [25]. The arrows are directed at the trypsin cleavage (R_36_-S_37_) and elastase cleavage (S_67_-V_68_) sites, up- and downstream of the tethered ligand [TL; the red colored letters (SLIGKV)] in the PAR2 N-terminal extracellular domain, respectively. **B** Nluc release assays from CHO-K1 cells expressing Nluc-hPAR2-eYFP were performed following treatment with increasing concentrations of L-asparaginase, trypsin or thrombin as described in “Materials and methods” section. Increase of luciferase activity due to the release of Nluc via proteolytic cleavage of the receptor was measured by Spectra iDX3 plate reader as described in “Materials and methods” section. Values in the left ordinate axes (), which are relative luminescence intensities in arbitrary units (AU), represent means ± SEM of the three independent experiments. Values in the right ordinate axes (), which are relative eYFP fluorescence intensities [in arbitrary units (AU)] at λ_ex_ = 510 _nm_ and λ_em_ = 535 _nm_, represent means ± SEM of three independent experiments. *p < 0.05.

To substantiate this observation, CHO-K1 cells expressing Nanoluciferase (Nluc)-PAR2-eYFP [25] were subjected to Nluc release assays to monitor the ability of L-asparaginase to cleave PAR2 N-terminal extracellular domain (Fig. 4A). To do so, cells suspended in HBSS were incubated with L-asparaginase and resulting supernatants, which contain Nluc released from proteolytic cleavage of the receptor, were examined for luciferase activity upon addition of its substrate, coelenterazine. As shown in Fig. 4B, L-asparaginase triggered dose-dependent increase of luminescence (left y-axes, ) with no significant increase of eYFP signal (right y-axes, ), reflecting the Nluc release. Trypsin-treated cells were used as positive control (Fig. 4B upper right panel). Cells treated with thrombin, which stimulates PAR1 but not PAR2, were used as negative control (Fig. 4B lower panel). Taken together, our findings indicate that L-asparaginase cleaves PAR2 at an unknown site(s) in the N-terminal extracellular domain.

### L-asparaginase cleaves N_30_-R_31_ and R_31_-S_32_ residues, upstream of the TL

**Fig. 5:**
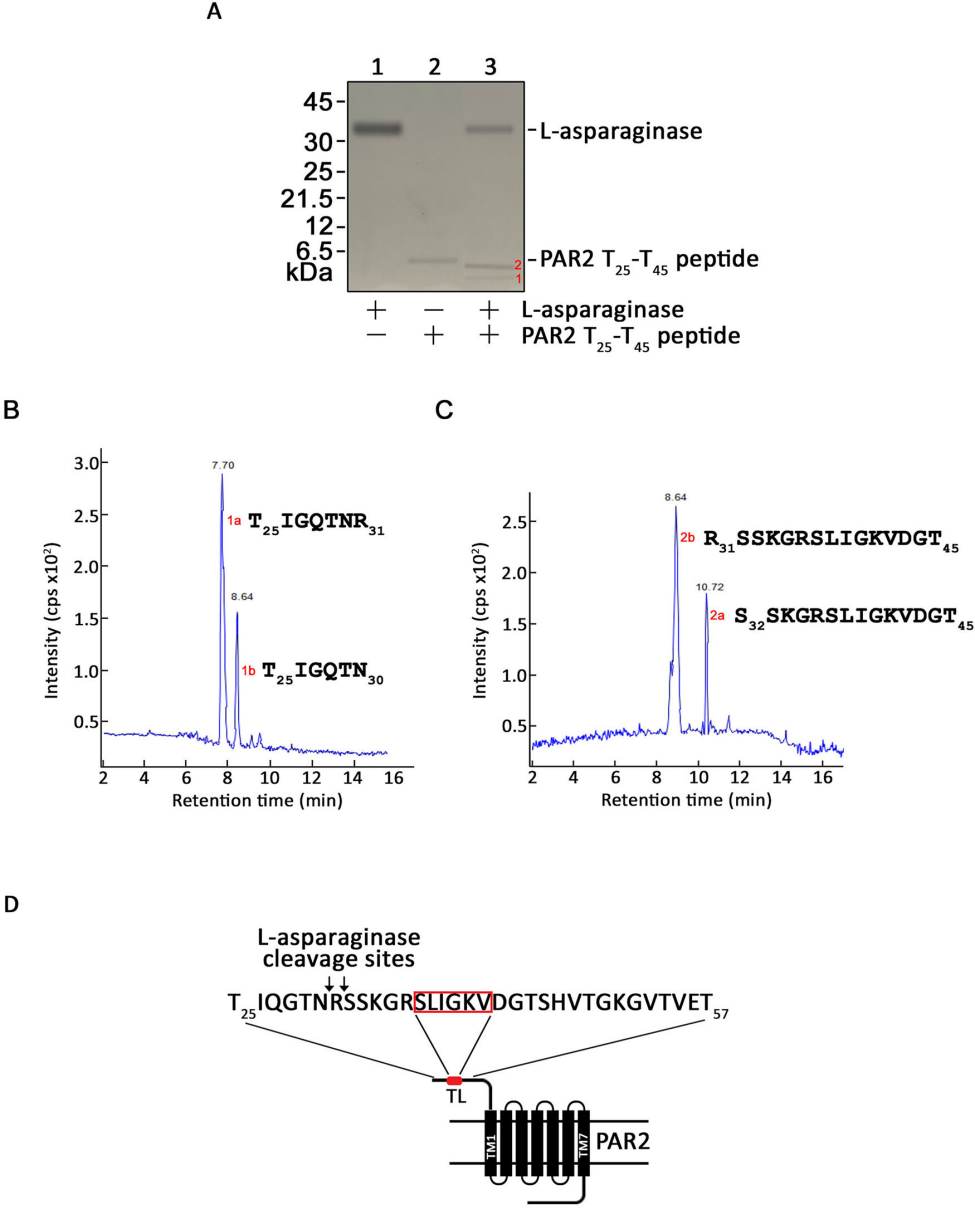
L-asparaginase cleaves N_30_-R_31_ and R_31_-S_32_ residues at the N-terminal extracellular domain. **A** 16% Tricine-SDS-PAGE analysis of PAR2 N-terminal T_25_-T_45_ peptide incubated with L-asparaginase and stained with Coomassie Brilliant blue R250 showed two cleaved fragments, designated as 1 and 2 (lane 3). Lane 1, L-asparaginase (5 µg); lane 2, T_25_-T_45_ peptide (0.5 µg) and land 3, L-asparaginase (2 µg) and T_25_-T_45_ peptide (0.5 µg) were used. LC analyses of the cleaved fragment 1 and 2 (**A**, lane 3) produced two peaks each at 7.70 (1a) and 8.64 (1b) min (**B**) and at 10.72 (2a) and 8.64 (2b) min (**C**), respectively. The subsequent tandem MS analyses revealed the sequences of 1a and 1b to be T_25_IGQTNR_31_ and T_25_IGQTN_30_ (**B**), respectively, that perfectly paired with the sequences of 2a and 2b, S_32_SKGRSLIGKVDGT_45_ and R_31_SSKGRSLIGKVDGT_45_ (**C**), respectively. **D** Schematic diagram of cell membrane PAR2. Arrows are directed at the L-asparaginase cleavage sites, N_30_-R_31_ and R_31_-S_32_, upstream of the tethered ligand (TL; SLIGKV, boxed in red) in the N-terminal extracellular domain. N-terminal extracellular domain, seven transmembrane domains (black bars 1–7), three extracellular loops, three intracellular loops and an intracellular C-terminal domain are also shown.

To identify L-asparaginase cleavage site(s) in the PAR2 N-terminal extracellular domain, a PAR2 T_25_-T_45_ peptide, which contains the known cleavage site (R_**36**_-S_**37**_) of trypsin just upstream of the TL, was synthesized and treated with L-asparaginase or trypsin (as positive control). The resulting peptide fragments were resolved in a 16% tricine SDS-PAGE. As shown in Supplementary Fig. 1A, trypsin digestion of the peptide produced two fragments (1 and 2; Supplementary Fig. 1A, lane 3). LC analysis of the fragmented peptides 1 and 2 (Supplementary Fig. 1A, lane 3) showed a peak eluted at 6.19 (Supplementary Fig. 1B) and 5.93 (Supplementary Fig. 1C) min, respectively. Subsequent tandem MS (MS/MS) analyses revealed the sequences of the fragmented peptides 1 and 2 to be T_25_IQGTNRSSKGR_36_ (Supplementary Fig. 1B) and S_37_LIGKVDGT_45_ (Supplementary Fig. 1C), respectively, indicating that trypsin indeed cleaved the T_25_-T_45_ peptide at R_**36**_-S_**37**_ (Supplementary Fig. 1D). After confirming that our approach for identification of the trypsin cleavage site using the PAR2 T_25_-T_45_ peptide was accurate, we proceeded to identify L-asparaginase cleavage site(s) in the PAR2 T_25_-T_45_ peptide. As shown in Fig. 5, L-asparaginase also produced two fragments (1 and 2; Fig. 5A, lane 3). LC analyses of fragmented peptides 1 and 2 (Fig. 5A, lane 3) produced two peaks each at 7.70 (1a) and 8.64 (1b) min (Fig. 5B) and at 10.72 (2a) and 8.64 (2b) min (Fig. 5C), respectively. Our subsequent MS/MS analyses uncovered the sequences of 1a and 1b to be T_25_IGQTNR_31_ and T_25_IGQTN_30_ (Fig. 5B), respectively, which perfectly paired with the sequences of 2a and 2b, S_32_SKGRSLIGKVDGT_45_ and R_31_SSKGRSLIGKVDGT_45_ (Fig. 5B), respectively. These findings indicate that L-asparaginase cleaves two distinct sites in the PAR2 N-terminal extracellular domain: N_30_-R_31_ and R_31_-S_32_, both of which are located upstream of the TL. Thus, L-asparaginase induces IP3R-mediated ER Ca^2+^ release (Fig. 1) by cleaving these two sites that could expose the TL for PAR2 activation.

**Fig. 6:**
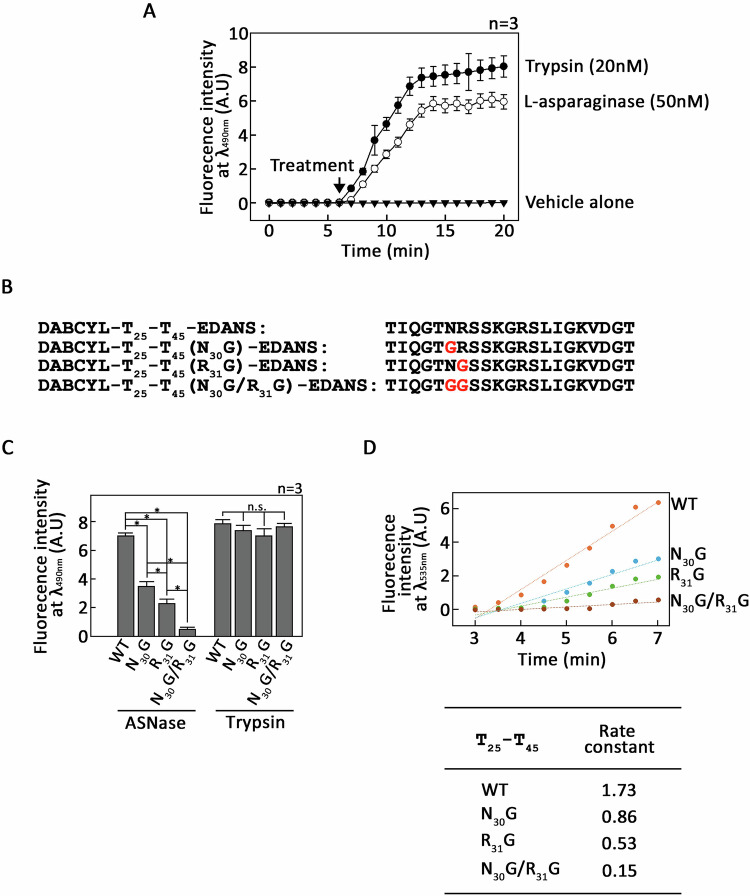
FRET-based L-asparaginase cleavage assays of wt-, N_30_G-, R_31_G- and N_30_G/R_31_G-substituted forms of DABCYL-PAR2 T_25_-T_45_-EDANS indicate that L-asparaginase specifically cleaves N_30_-R_31_ and R_31_-S_32_ at the N-terminal extracellular domain. **A** DABCYL-PAR2 T_25_-T_45_-EDANS incubated with L-asparaginase (○) or trypsin (positive control; ●) for the indicated time periods were subjected to spectrofluorometry using an IDX3 Spectramax (Molecular Devices). Values, which are relative fluorescence intensities at λ_ex_ = 340 _nm_ and λ_em_ = 490 _nm_ in arbitrary units (AU), represent means ± SEM of the three independent experiments (*n* = 3). **B** Amino acid sequences of wt-, N_30_G-, R_31_G- and N_30_G/R_31_G-substituted forms of DABCYL-PAR2 T_25_-T_45_-EDANS are shown. **C**. Wt-, N_30_G-, R_31_G- and N_30_G/R_31_G-substituted forms of DABCYL-PAR2 T_25_-T_45_-EDANS incubated with L-asparaginase were subjected to spectrofluorometry using IDX3 Spectramax (Molecular Devices). Trypsin, which cleaves the PAR2 T_25_-T_45_ peptide at R_**36**_-S_**37**_ (Supplementary Fig. 1D), was used as positive control. Values, which are relative fluorescence intensities at λ_ex_ = 340 _nm_ and λ_em_ = 490 _nm_ in arbitrary units (AU) after 14 min of L-asparaginase treatment, represent means ± SEM of three independent experiments (*n* = 3). *p < 0.05. **D** Wt-, N_30_G-, R_31_G- and N_30_G/R_31_G-substituted forms of DABCYL-PAR2 T_25_-T_45_-EDANS incubated with L-asparaginase for the indicated time period were subjected to spectrofluorometry using an IDX3 Spectramax (Molecular Devices). Increase in relative fluorescence intensities at λ_em_ = 490 _nm_ in arbitrary units (AU) were plotted as a function of time to calculate rate constants from the slope of the lines of best fit.

Next, the PAR2 T_25_-T_45_ peptide was labeled N-terminally with DABCYL, a quencher and C-terminally with EDANS, a fluorophore. Thus, the fluorescence of EDANS fluorophore in the intact peptide is quenched by the proximity of the DABCYL quencher via fluorescence resonance energy transfer (FRET). However, upon cleavage of DABCYL-PAR2 T_25_-T_45_-EDANS by L-asparaginase, DABCYL and EDANS are split, resulting in a rise of EDANS’ fluorescence, which can be detected by spectrofluorometer. As shown in Fig. 6A, L-asparaginase (○) noticeably increased EDANS’ fluorescence while the vehicle alone (▼) displayed negligible EDANS’ fluorescence. Trypsin, which cleaves the PAR2 T_25_-T_45_ peptide at R_**36**_-S_**37**_ (Supplementary Fig. 1D), was used as a positive control (Fig. 6A, ●). After establishing the FRET-based DABCYL-PAR2 T_25_-T_45_-EDANS cleavage assay for L-asparaginase, N_30_G, R_31_G and N_30_G/R_31_G substituted forms of DABCYL-PAR2 T_25_-T_45_-EDANS (Fig. 6B) were generated to substantiate the above findings. As shown in Fig. 6C, L-asparaginase-mediated increase in EDANS’ fluorescence was attenuated when N_30_G and R_31_G substituted forms of DABCYL-PAR2 T_25_-T_45_-EDANS were used. Substitutions of both sites resulted in almost complete inhibition of EDANS’ fluorescence upon L-asparaginase treatment. This validates the above finding that L-asparaginase indeed cleaves the N_30_-R_31_ and R_31_-S_32_ residues.

Our observation showing greater inhibition in L-asparaginase-mediated EDANS’ fluorescence when an R_31_G substituted form of DABCYL-PAR2 T_25_-T_45_-EDANS was used compared to N_30_G substituted forms of DABCYL-PAR2 T_25_-T_45_-EDANS led us to compare their rate constants. To do so, increases in relative fluorescence intensities of N_30_G, R_31_G and N_30_G/R_31_G substituted forms of DABCYL-PAR2 T_25_-T_45_-EDANS upon L-asparaginase treatment were plotted over time, and rate constants in EDANS’ fluorescence were calculated by producing lines of best fit to calculate the slope of these lines. As shown in Fig. 6D, we found that the N_30_G substituted peptide rate constant was faster than that of R_31_G substituted peptide, implying that compared to N_30_, R_31_ is the preferred target of L-asparaginase.

### The new extended TL peptides cause ER Ca^2+^ release

**Fig. 7:**
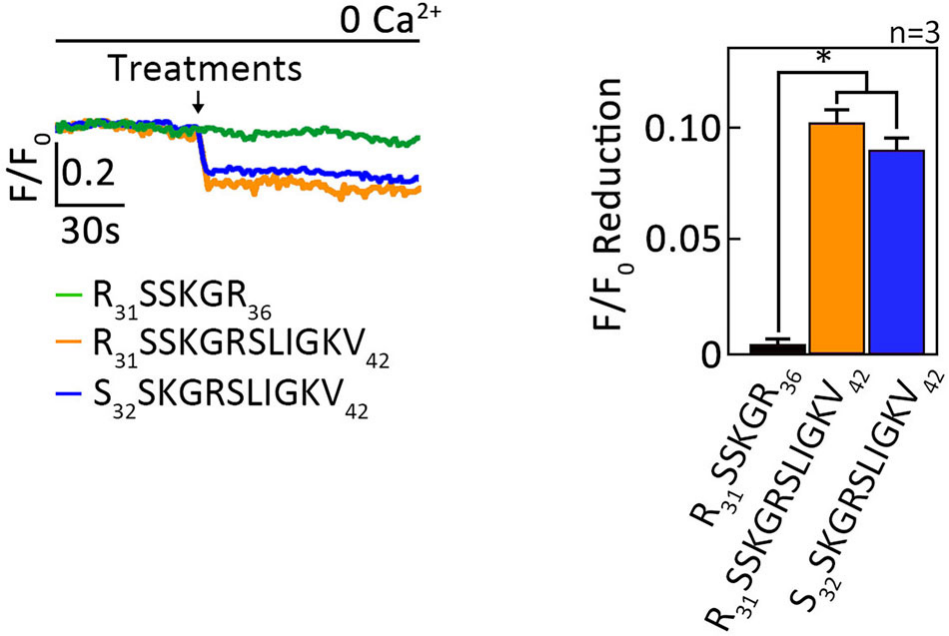
The new extended TL peptides induce ER Ca^2+^ release. µ-OR1-knockdown SEM cells loaded with Mag Fluo-4 AM were subjected to Ca^2+^ tracing by single-cell Ca^2+^ imaging. After obtaining stable baseline ER Ca^2+^ levels for 1 min, cells were treated with R_31_SSKGR_36_, R_31_SSKGRSLIGKV_42_ or S_32_SKGRSLIGKV_42_ for 1.5 min to analyze ER Ca^2+^ release. The left panel shows the average Ca^2+^ tracing measured every two seconds in 20 individual cells after R_31_SSKGR_36_, R_31_SSKGRSLIGKV_42_ or S_32_SKGRSLIGKV_42_ treatment. Data are from one of three independent experiments (*n* = 3) showing similar results. The charts on the right show the difference in ER Ca^2+^ release following treatment with R_31_SSKGR_36_, R_31_SSKGRSLIGKV_42_ or S_32_SKGRSLIGKV_42_. *F*/*F*_0_ value of 1 min after R_31_SSKGR_36_, R_31_SSKGRSLIGKV_42_ or S_32_SKGRSLIGKV_42_ was used to determine *F*/*F*_0_ reduction. Values are means ± SEM of the three independent experiments *p < 0.05.

Since L-asparaginase cleavage of PAR2 N-terminal N_30_-R_31_ and R_31_-S_32_ residues creates new extended TLs (R_31_SSKGRSLIGKV_42_ and S_32_SKGRSLIGKV_42_), we examined whether L-asparaginase activates PAR2 by exposure of these new extended TLs. To do so, we synthesized R_31_SSKGRSLIGKV_42_ and S_32_SKGRSLIGKV_42_. R_31_SSKGR_36_ was used as negative control. As shown in Fig. 7, the new extended TLs peptides caused ER Ca^2+^ in µ-OR1-knockdown SEM aLL cells, while the control peptide, which has the same amino acid sequence as the extended TLs but lacks the TL residue (S_37_LIGKV_42_), did not. Thus, this finding indicates that L-asparaginase-driven PAR2-dependent ER Ca^2+^ release is due to exposure of new extended TLs.

## Discussion

The fact that PAR2 is activated by a serine protease, trypsin, which cleaves PAR2 N-terminal R_**36**_-S_**37**_ residue, exposing the TL (S_37_LIGKV_42_) [11, 26], and our recent finding [9] that L-asparaginase, which has β-aspartyl peptidase activity [13–18], prompts IP3R-mediated ER Ca^2+^ release by targeting PAR2 led us to investigate whether L-asparaginase also targets the PAR2 N-terminal extracellular domain. If this holds true, we anticipated that pretreatment with elastase should abolish L-asparaginase-induced PAR2-mediated ER Ca^2+^ release like elastase did to trypsin-induced PAR2-mediated ER Ca^2+^ release [20]. Indeed, we demonstrated inhibition of L-asparaginase-induced ER Ca^2+^ release in cells pretreated with elastase, indicating that PAR2 activation by L-asparaginase is probably mediated by TL exposure upon PAR2 N-terminal cleavage. The use of inactive forms of L-asparaginase carrying T_111_V/K_184_T or D_112_T/K_184_T substitution and µ-OR1-knockdown cells allowed us to show that L-asparaginase stimulation of PAR2 involves its enzymatic activity that cleaves the PAR2 N-terminal extracellular domain.

The N-terminal mRFP-release assays using HEK293T cells expressing mRFP-PAR2-eYFP by time-lapse confocal microscopy provided visual evidence that L-asparaginase cleaves the mRFP-PAR2 N-terminal extracellular domain off at the cell periphery over time. This observation was further substantiated by Nluc report release assays in CHO-K1 cells expressing Nluc-PAR2-eYFP that revealed dose-dependent increase of Nluc luminescence due to L-asparaginase-mediated cleavage of Nluc.

Since it has been demonstrated that asparaginyl (N) or aspartyl (D) peptides can be deaminated or dehydrated, respectively, under physiological conditions to form a cyclic imide intermediate that when hydrolyzed, forms β-aspartyl peptides that can be cleaved by L-asparaginase’s β-aspartyl peptidase activity [18], we investigated whether the PAR2 N-terminal extracellular domain has asparagine and/or aspartic acid residue(s). Indeed, we found that the PAR2 N-terminal extracellular I_26_-G_71_ domain contained an asparagine (N_30_) and two aspartic acids (D_43_ and D_62_) that can form β-aspartyl peptides, which may then be cleaved by L-asparaginase. However, L-asparaginase involvement of cleavage through the latter two aspartic acids (D_43_ and D_62_) is unlikely as they lie downstream of TL and the cleavage of either sites will disarm PAR2, counteracting PAR2-mediated ER Ca^2+^ release upon L-asparaginase treatment. Thus, to identify the L-asparaginase cleavage site(s) in the PAR2 N-terminal extracellular domain, a PAR2 T_25_-T_45_ peptide, which contains the potential L-asparaginase cleavage site (N_**30**_-R_**31**_) and the known cleavage site (R_**36**_-S_**37**_) of trypsin, a PAR2 agonist protease, was synthesized. L-asparaginase digestion of the PAR2 T_25_-T_45_ peptide and subsequent LC-tandem MS analyses showed that L-asparaginase cuts two different sites in the PAR2 N-terminal extracellular domain: N_30_-R_31_ and R_31_-S_32_, both of which are situated upstream of the TL. Therefore, this finding indicates that L-asparaginase elicits IP3R-mediated ER Ca^2+^ release by cleaving these two sites that unmask the TL for PAR2 activation.

To validate the L-asparaginase cleavage sites, we designed a FRET-based L-asparaginase cleavage assay using wt-, N_30_G-, R_31_G- and N_30_G/R_31_G-substituted PAR2 T_25_-T_45_ peptides that were tagged N-terminally with DABCYL, a quencher, and C-terminally with EDANS, a fluorophore. In this assay, cleavage of the dual tagged PAR2 T_25_-T_45_ by L-asparaginase can be detected by a rise of EDANS’ fluorescence as a result of the EDANS fluorophore and DABCYL quencher breakup. Our data showing partial and complete inhibition of L-asparaginase-mediated increase in EDANS’ fluorescence when individual (N_30_G or R_31_G) and combined (N_30_G and R_31_G) substituted form of DABCYL-PAR2 T_25_-T_45_-EDANS were used, respectively, suggest that L-asparaginase targets the two N_30_ and R_31_ residues in the PAR2 N-terminal extracellular domain. Indeed, asparagine deamidation is one of the most common modifications that spontaneously occurs without enzymatic reaction in proteins and peptides [18]. Interestingly, it was shown that legumain, which was originally purified and characterized from legume seeds [27], cleaves PAR2 extracellular N-terminal N_30_-R_31_ via its legumain’s asparaginyl endopeptidase activity and activates PAR2, increasing [Ca^2+^]_i_ [28].

Our finding that the rate constant of the N_30_G substituted peptide in EDANS’ fluorescence was greater than that of R_31_G substituted peptide implies that compared to N_30_, R_31_ is the preferred target of L-asparaginase. To date, there has been no report indicating that L-asparaginase targets arginine. However, arginine can be converted into citrulline by arginine deaminase [29] and subsequent deamination could form a cyclic imide intermediate that may be targeted by L-asparaginase’s aspartyl peptidase activity. Our data showing inhibition of L-asparaginase-mediated increase in EDANS’ fluorescence when R_31_G substituted form of DABCYL-PAR2 T_25_-T_45_-EDANS was used is in consistent with this view.

Although it has been over two decades since L-asparaginase was known to have β-aspartyl peptidase activity [13–18], its biological significance remains poorly understood. Thus, our finding showing that L-asparaginase stimulates PAR2 by cleaving N_30_-R_31_ and R_31_-S_32_ residues and unmasking the new extended TLs, establishes the first example relating its β-aspartyl peptidase with the regulation intracellular Ca^2+^ homeostasis via PAR2 stimulation, that underlies the ability of L-aspariginase to induce apoptosis of aLL cells in cancer patients.

## Materials and methods

### Materials

Dulbecco’s modified Eagle medium (DMEM)/F12, DMEM and RPMI1640 were from Thermo Fischer (ON, Canda). JetPRIME transfection reagent was from Polyplus transfection (NY, USA). L-asparaginase (ab277068) was from Abcam (ON, Canada). Coelenterazine was from Promega (ON, Canada). Coomassie Brilliant blue R250, thrombin, trypsin, poly-L-ornithine, tert-butylhydroquinone (TBHQ) and hydrocortisone were from Sigma-Aldrich (ON, Canada). L-glutamine, fetal bovine serum (FBS), penicillin/streptomycin, puromycin and HEPES (4-(2-hydroxyethyl) piperazine-1-ethanesulfonic acid were from Gibco (CA, USA). G418 was from Invitrogen (MA, USA). PAR2 agonist and 2-furoyl-LIGRLO-NH_2_ were synthesized by FMOC solid phase peptide synthesis (SPPS) method at the University of Calgary Peptide Synthesis Facility. PAR2 T_25_-T_45_ (TIQGTNRSSKGRSLIGKVDGT), R_31_SSKGR_36_, R_31_SSKGRSLIGKV_42_, S_32_SKGRSLIGKV_42_ were synthesized through GenScript (NJ, USA). These peptides were >95% pure products, verified by HPLC and mass spectrometry.

### Cell culture

SEM aLL cell line [6] originally established from a female 5-year-old patient diagnosed with B-cell aLL and HEK293T human embryonic kidney cells were cultured in RPMI1640 and DMEM, respectively, at 37°C and CO_2_ level of 5%. CHO-K1 cells were cultured in DMEM/F12 containing 25 mM HEPES, 1 µg/ml hydrocortisone and 2 mM L-glutamine at 37°C and CO_2_ level of 5%. The media were supplemented with 10% fetal bovine serum, and 100 μg/ml penicillin/streptomycin. SEM cells stably transduced with lentivirus carrying sh*µ-OR1* shRNA were generated [9] according to the manufacturer’s instructions. Briefly, SEM cells grown to 50–60% confluence in 6-well plates were infected with lentivirus carrying sh*µ-OR1* shRNA in the presence of 5 µg/ml polybrene followed by four consecutive passages of 10 μg/ml puromycin-selected cells [9]. Cells were tested for mycoplasma contamination.

### Measurement of endoplasmic reticulum (ER) Ca^2+^ release

SEM (0.5×10^6^) grown on 0.2 mg/ml poly-L-ornithine-coated 12 mm glass coverslips were loaded with 2 µM Mag-Fluo-4 AM in RPMI media for 45 min. Coverslips were then transferred to a 3.5 cm glass bottom plate containing 1 ml of Ca^2+^-free Krebs-Ringer-Henseleit (KRH) buffer (25 mM HEPES, pH 7.4, 125 mM NaCl, 5 mM KCl, 6 mM glucose, 1.2 mM MgCl_2,_ and 2 μM EGTA). Ca^2+^ transients were traced using the DMi8-Film microscope at a magnification of 20x (λ_Ex_ = 495 _nm_ and λ_Em_ = 530 _nm_) and the LASX imaging software (Leica Microsystems). After obtaining stable baseline ER Ca^2+^ levels for 1 min, cells were pretreated (or not pretreated) with 10 units/ml elastase for 1 min, then treated with 100 mIU/ml L-asparaginase, 100 nM trypsin, 2 µM 2-furoyl-LIGRLO-NH_2_, 1.5 µM R_31_SSKGR_36_, 1.5 µM R_31_SSKGRSLIGKV_42_, 1.5 µM S_32_SKGRSLIGKV_42_ for the indicated period of time followed by 10 µM TBHQ. The average Ca^2+^ tracings were measured per sec in 10-20 individual cells following the treatment. The resulting ER Ca^2+^ release was assessed through changes in ER fluorescence: the signal-to-baseline ratio (SBR), which is simply the F/F_0_ ratio, where F= fluorescence value after stimulation and F_0_= basal or initial fluorescence).

### Purification of wt and T_111_V/K_184_T or D_112_T/K_184_T substituted forms of L-asparaginase

*Escherichia coli* JC2 lacking three genes (*ansA*, *ansB* and *iaaA*) encoding endogenous asparaginases but harboring pET22b(+) vector containing wt or T_111_V/K_184_T or D_112_T/K_184_T substituted form of L-asparaginase [22–24] was provided by Dr. Sergei Sukharev at the University of Maryland. These clones were originally from Drs. Philip Lorenzi and John Weinstein’s laboratory at MD Anderson. L-asparaginases were purified following the procedure established by Lubkowski et al [22]. Briefly, after overnight induction at 37 °C in LB + 50 µg/ml ampicillin, L-asparaginases secreted in the media were clarified by centrifugation at 8000 × *g* for 15 min, filtered using 0.22 µm membrane filter (Millipore, ON, CA), mixed with 0.5 mM PMSF, 0.3 M NaCl and His60 Ni Superflow resin, and incubated in a rocker for 3 h at 4 °C. The mixture was loaded into a gravity column and washed with 50 mM phosphate buffer (pH 7.4) containing 0.5 mM PMSF, 0.3 M NaCl and 20 mM imidazole. L-asparaginases were eluted with 0.5 M imidazole then dialyzed using Spectra/Por® 8,000 for 12 h at 4 °C. To assess for purity, the resulting proteins were analyzed by 12.5% SDS-PAGE and staining with 0.1% Coomassie brilliant blue R250.

### Measurement of L-asparaginase activity

The activity of the wt EcA2 and its mutant forms (T_111_V/K_184_T and D_112_T/K_184_T) was assessed by monitoring the enzyme-coupled oxidation of NADH to NAD^+^ as described by Fernandez et al. [30]. Briefly, a reaction mixture containing 20 mg of α-ketoglutarate, 26.4 mg of L-asparagine, 120 units of glutamic oxaloacetic transaminase, 20 mg of NADH, and 200 units of malic dehydrogenase in 200 ml of TBS (pH 7.4) with 20% glycerol was prepared. L-asparaginase activity was measured by adding 10 µl of wt or mutant L-asparaginase (2 µg/µl) to 190 µl of the reaction mixture and monitoring the absorbance of NADH at 340 nm every 30 s at 37 °C on a SpectraMax iD3 microplate reader (Molecular devices, Wals, Austria).

### Visualization of L-asparaginase cleavage of PAR2

HEK293T cells stably transfected with pcDNA3.1(+) construct carrying monomeric red fluorescent protein (mRFP)-human PAR2 (hPAR2)-enhanced yellow fluorescent protein (eYFP) [25] using JetPRIME transfection reagent/800 µg/ml G418 were grown on 0.2 mg/ml poly-L-ornithine-coated 12 mm glass coverslips in 12-well plate for 24 h. Coverslips transferred to 3.5 cm glass bottom dish were treated with 100 mIU of L-asparaginase or 50 nM trypsin for the indicated time periods. Release of mRFP signal was monitored by Zeiss LSM 710 time-lapse confocal microscope (Zeiss, Germany) at ×40 magnification.

### Luciferase assay

CHO-K1 cells (1 × 10^5^/well) stably transfected with Nanoluciferase (Nluc)-hPAR2-eYFP [25] using JetPRIME transfection reagent/800 µg/ml G418 were plated in a 24-well plate and cultured for 24 h in DMEM/F12 complete medium. Cells were then rinsed thrice with 100 µl of HBSS and incubated with the indicated concentrations of L-asparaginase, trypsin or thrombin at 37 °C up to 20 min. Fifty μls of the resulting supernatant/media were transferred to a 96-well white flat bottom microplate, added 2 µM coelenterazine, and incubated for 15 min. Increase of luciferase activity of Nluc, due to the release of Nluc from proteolytic cleavage of the receptors, was measured by Spectra iDX3 plate reader (Molecular Device, CA, USA) as described previously [31].

### Identification of L-asparaginase cleavage sites

PAR2 T_25_-T_45_ (0.5 µg) treated with 1 µg trypsin or 2 µg L-asparaginase was resolved by 16% Tricine-SDS-PAGE and subsequently stained with Coomassie Brilliant blue R250. Cleaved fragments excised out from the gel were recovered by passive extraction with 0.1% SDS/100 mM ammonium bicarbonate followed by desalting using methanol/chloroform/water precipitation and reconstitution in 5 μl of 0.1% TFA. LC-tandem MS(MS/MS) of the fragments was carried out through the Alberta Proteomics and Mass Spectrometry Facility at the University of Alberta. Briefly, the fragments were subjected to nano-flow HPLC (Easy-nLC 1000, Thermo Scientific) coupled to a Q Exactive Orbitrap mass spectrometer (Thermo Scientific) with an EASY-Spray capillary HPLC column (ES800A, Thermo Scientific). The mass spectrometer was operated in data-dependent acquisition mode, recording high-accuracy and high-resolution survey orbitrap spectra using external mass calibration, with a resolution of 35,000 and an m/z range of 200–1500. The 12 most intense multiply charged ions were sequentially fragmented using high-energy collision-induced dissociation, and the spectra of their fragments were recorded in the orbitrap at a resolution of 17,500. Data were processed using Proteome Discoverer 1.4 (Thermo Scientific), and the database was searched using SEQUEST (Thermo Scientific). Search parameters included a strict false discovery rate (FDR) of 0.01, a relaxed FDR of 0.05, a precursor mass tolerance of 10 ppm, and a fragment mass tolerance of 0.01 Da. Peptides were searched with carbamidomethyl cysteine as a static modification and oxidized methionine and deamidated glutamine and asparagine as dynamic modifications.

### Cleavage assays for DABCYL-PAR2 T_25_-T_45_-EDANS

DABCYL-PAR2 T_25_-T_45_-EDANS, designated as DABCYL-PAR2 T_25_-T_45_-EDANS, and N_30_G, R_31_G and N_30_G/R_31_G substituted forms were synthesized through GenScript (NJ, USA) as >95% pure products, verified by HPLC and mass spectrometry. WT and N_30_G, R_31_G and N_30_G/R_31_G substituted forms of DABCYL-PAR2 T_25_-T_45_-EDANS incubated with 50 nM L-asparaginase or 20 nM trypsin were subjected to spectrofluorometry using IDX3 Spectramax (Molecular Devices) at λ_ex_ = 340 _nm_ and λ_em_ = 490 nm.

### Statistical analysis

The student’s unpaired, two-tailed *t*-test was performed with *p* < 0.05 being considered statistically significant.

## Supplementary information

Supplementary Figure 1

Supplementary Figure legend

## Data Availability

All data generated or analyzed during this study are included in this published article and its supplementary information files/uncropped data files.
