# Supplementary figures and images for: L-asparaginase is a PAR2 N-terminal protease that unmasks the PAR2 tethered ligand
Source: Cell Death Discov. 2025 Apr 8;11:152. doi: 10.1038/s41420-025-02467-z (PMC11977020; doi:10.1038/s41420-025-02467-z)

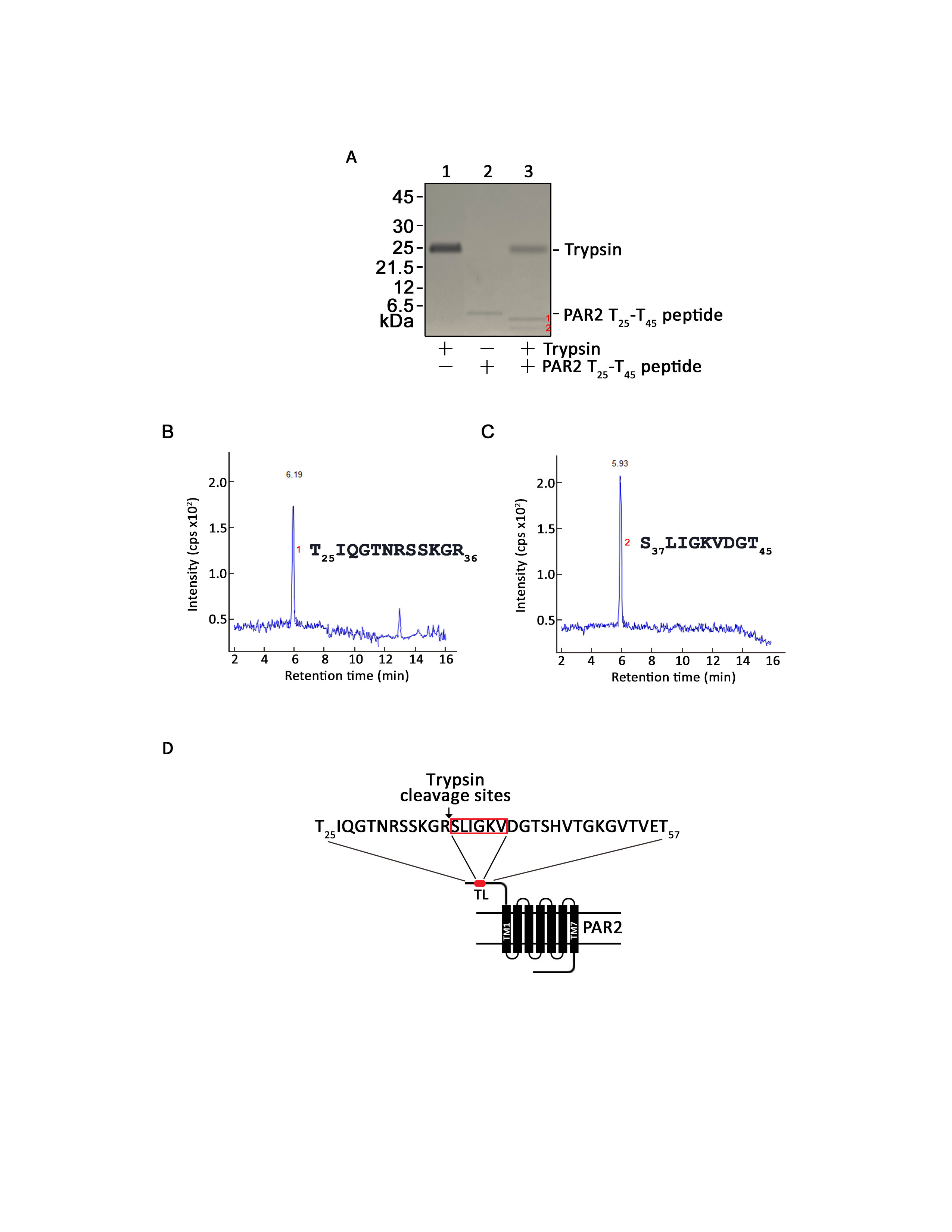

Supplement: Supplementary file 1 — Supplementary Figure 1 [file 41420_2025_2467_MOESM1_ESM.jpg]
