# Supplementary material for: L-asparaginase is a PAR2 N-terminal protease that unmasks the PAR2 tethered ligand
Source: Cell Death Discov. 2025 Apr 8;11:152. doi: 10.1038/s41420-025-02467-z (PMC11977020; doi:10.1038/s41420-025-02467-z)
Supplement: Supplementary file 2 — Supplementary Figure legend [file 41420_2025_2467_MOESM2_ESM.docx]

**Supplementary Figure legend**

**Supplementary Figure 1. Trypsin cleaves the R_36_-S_37_ residue at the N-terminal extracellular domain**. **A**. 16% Tricine SDS-PAGE analysis of PAR2 N-terminal T_25_-T_45_ peptide incubated with trypsin and stained with Coomassie Brilliant blue R250 shows two cleaved fragments, designated as 1 and 2 (lane 3). Lane 1, trypsin (4 µg); lane 2, T_25_-T_45_ peptide (0.5 µg) and lane 3, trypsin (1 µg) and T_25_-T_45_ peptide (0.5 µg) were used. **B & C**. LC analyses of the cleaved fragment 1 and 2 (A, lane 3) showed a peak eluted at 6.19 (B) and 5.93 min (C), respectively. Subsequent MS/MS analyses revealed sequences of the cleaved fragments 1 and 2 to be T_25_IQGTNRSSKGR_36_ (**B**) and S_37_LIGKVDGT_45_ (**C**), respectively. **D**. Schematic diagram of cell membrane PAR2. The arrow is directed at the trypsin cleavage site, R**_36_**-S**_37_**, upstream of the tethered ligand (TL; SLIGKV, boxed in red) in the PAR2 N-terminal extracellular domain. N-terminal extracellular domain, seven transmembrane domains (black bars 1-7), three extracellular loops, three intracellular loops and an intracellular C-terminal domain are also shown.
